# Histoplasmosis in HIV-Infected Patients: A Review of New Developments and Remaining Gaps

**DOI:** 10.1007/s40475-014-0017-8

**Published:** 2014-03-28

**Authors:** Antoine A. Adenis, Christine Aznar, Pierre Couppié

**Affiliations:** 1 Inserm CIC 1424, Centre d’Investigation Clinique Antilles-Guyane, Centre Hospitalier de Cayenne, avenue des flamboyants, BP 6006, 97 300 Cayenne, France; 2 UAG EA 3593, Epidémiologie des Parasitoses et des Mycoses Tropicales, Université des Antilles et de la Guyane, Cayenne, France; 3 Laboratoire Hospitalo-Universitaire de Parasitologie-Mycologie, Centre Hospitalier de Cayenne, Cayenne, France; 4 Service de Dermatologie Vénérologie,, Centre Hospitalier de Cayenne, Cayenne, France

**Keywords:** HIV, AIDS, Histoplasmosis, *Histoplasma*, Tropical medicine, Fungal disease, Tropical mycosis

## Abstract

*Histoplasma capsulatum* is responsible for histoplasmosis, a fungal disease with worldwide distribution that can affect both immunocompromised and imunocompetent individuals. During the highly active antiretroviral therapy (HAART) era, morbidity and mortality due to histoplasmosis remained a public heatlh problem in low-income and high-income countries. The true burden of HIV-associated histoplasmosis is either not fully known or neglected since it is not a notifiable disease. Progress has been made in DNA patterns of strains and understanding of pathogenesis, and hopefully these will help identify new therapeutic targets. Unfortunately, histoplasmosis is still widely mistaken for multidrug-resistant tuberculosis, leading to numerous avoidable deaths, even if they are easily distinguishable. The new diagnostic tools and therapeutics developments have still not been made available in most endemic regions. Still, recent developments are promising because of their good clinical characteristics and also because they will be commercially available and affordable. This review of published data and gaps may help define and guide future research.

## Introduction

*Histoplasma capsulatum* (*H. capsulatum*) is the etiologic agent of histoplasmosis, a fungal disease that can affect both immunocompromised and imunocompetent individuals. It was first described in 1905 by S.T. Darling, concerning a case that looked like disseminated tuberculosis in a patient originated from Martinique who was working at building the Panama Canal [1].

Since then, histoplasmosis was described on five continents, with high and low endemicity areas. Thus, epidemiologic, clinical, paraclinical and therapeutic data are available and have led to numerous publications by reference centers, based on autochthonous cases in endemic areas [2–7] or imported cases in non-endemic areas [8–10].

Labeled as the first respiratory fungal infection by some authors, the public health burden of histoplasmosis differs depending on whether the person is immunocompromised or not. Among immunocompetent persons, apart from histoplasmin test campaigns, few serologic prevalence studies have been conducted in the general population. However, some authors estimate that hundred of thousands cases occur every year in the Ohio and Mississipi River regions. Mostly asymptomatic and spontaneously self-limited, histoplasmosis causes low morbidity and mortality among immunocompetent patients [11].

Among immunocompromised patients, in the presence of an acquired or congenital cellular immunity deficiency, histoplasmosis is responsible for an important morbidity and mortality. At all ages, it is mostly fatal in the absence of appropriate treatment. Following the first cases described in 1982 in human immunodeficiency virus (HIV) infected patients in the USA, its extrapulmonary or disseminated form became an acquired immunodeficiency syndrome (AIDS)-defining infection in 1987 [12, 13].

During the last decades, morbidity and mortality from histoplasmosis have increased because of the increase in the number of immunocompromised persons. Although immunosuppressors used in transplant patients or chronic inflammatory diseases contribute to this increase, the greater part of the problem is mostly attributable to the spread of the HIV pandemic. [14]. The availability of highly active antiretroviral therapy (HAART) and lipid formulations of amphotericin B, the increased awareness of the problem by some teams, and the development of rapid, noninvasive, diagnostic methods have led to a major impact on mortality by histoplasmosis [15]. This progress was so spectacular that some authors no longer consider histoplasmosis a problem in the course of the HIV infection, considering instead that research should focus on other causes of immunosuppression associated with histoplasmosis [11, 16].

However, these developments are heterogeneous, and some authors consider HIV-associated histoplasmosis a neglected disease, notably in South America [17]. Evolving under the radar of health authorities and research institutions, it is thought to have caused several thousand deaths in the Amazon region alone since the beginning of the epidemic [15].

Independently of the availability of HAART, histoplasmosis in HIV-infected patients remains a public health problem that is overlooked in numerous countries in endemic areas, notably in South America. To tackle this problem, there are important needs in terms of epidemiological knowledge, health professionals becoming aware of it, diagnostic development and availability of effective treatments at affordable costs [17].

This review will consider the recent developments and gaps regarding the epidemiological, diagnostic (clinical and biological) and therapeutic aspects of histoplasmosis in HIV-infected patients. The objective is to describe the current situation in order to guide future research.

## Taxonomy and Molecular Epidemiology

Historically, *H. capsulatum* was considered to be divided into three varieties based on morphologic characteristics: *H. capsulatum* var. *capsulatum*, mainly described in the Americas; *H. capsulatum* var. *duboisii*, mainly described in Africa; and *H. capsulatum* var. *farciminosum*, described in equines from Africa and the Middle East [18].

The *H. capsulatum* species is not monophyletic and has been recently subdivided into geographically distinct phylogenetic lineages [19]. Based on concordance of multiple gene sequence genealogies, *Histoplasma* strains separate into at least eight clades: North American class 1 (Nam1), North American class 2 (Nam2), Latin American group A (LamA), Latin American group B (LamB), Australian, Netherlands (Indonesian), Eurasian, and African clades [20]. Seven of these eight clades comprise genetically and geographically distinct populations that can be regarded as phylogenetic species. The single exception, the Eurasian clade, originated from within the Latin American group A clade. *H. capsulatum* var. *farciminosum* was placed within the Eurasian clade. In addition to the seven phylogenetic species, another seven lineages represented by single isolates from Latin America were identified [18, 20].

Interestingly, clinical differences in histoplasmosis disease manifestation have been reported among the groups [19]. At this time, histoplasmosis due to *H. capsulatum* var. *duboisii* was considered a distinct entity, causing primarily cutaneous and subcutaneous lesions rather than pulmonary involvement. Unlike var. *capsulatum* and according to published data, var. *duboisii* is still not considered as an AIDS-defining illness [21]. Whether this manifestation is determined by genetic differences in *Histoplasma* strains is unclear, since the taxonomic placement of *H. capsulatum* var. *duboisii* has been called into question by the finding of one var. *capsulatum* isolate from South Africa (primarily causing pulmonary disease) that was placed in the African clade (var. *duboisii*-containing) [19, 20]. This extends the results of earlier studies that had shown that var. *duboisii* had mitochondrial DNA restriction patterns identical to those of var. *capsulatum* strains [18]. In the Americas, authors have reported mucocutaneous tropism differences between North and South strains in HIV-infected patients as linked with genetic differences in *Histoplasma* strains [22, 23]. These findings didn’t reach consensus, since a marked decrease of mucocutaneous manifestation was observed by others during the HAART era, concomitant to the opening of a laboratory specialized in mycology [24, 25]. Other differences involving Nam1 and/or Nam2 and/or Latin American strains, concerning either the disease potential progression (acute or chronic) or the *Histoplasma* strain infection potential according to the immune status of the host (HIV-infected or not), have been described in the Americas with contradictory results [19].

Multiple typing methods have been developed to study the epidemiology of *H. capsulatum*. Nowadays, molecular typing methods are generally considered to have advantages over phenotypic methods in terms of stability of genomic markers and greater levels of type-ability. Several genotype-based methods, such as hybridization of target genes (probes), chromosomal DNA typing, restriction fragment length polymorphism (RFLP) analysis, random amplified polymorphic DNA (RAPD) analysis, and sequencing, have been described for *H. capsulatum* [26]. No single approach based on DNA assays has been the dominant method. Nevertheless, the analysis of concordance in the results obtained with different methods using the same set of isolates showed great convergence [26].

Thus, advances in the classification of *H. capsulatum* are sustained by the results of several genetic analyses that support the high diversity of this dimorphic fungus, suggesting diversity among strains in virulence, infectivity, pathogenesis and relationship to the immune status of the host [19]. But all these findings are based on relatively small sample size, and studies are required in order to explore the functional consequences of these variations on histoplasmosis pathogenesis in HIV-infected patients.

## Epidemiology

*H. capsulatum* is a fungus that is distributed worldwide, endemic to geographically limited areas, according to local environmental conditions favorable to its development [27]. Since histoplasmosis is not classified among the notifiable diseases, hard data on the incidence and prevalence, as well as information on its morbidity and mortality, should be considered fragmentary or simply not available in many endemic areas [28, 29].

It is admitted that histoplasmosis occurs most commonly in the Americas, since most of the studies are published from patients residing in this area. But the organism exists in many diverse areas around the world and is described in the non-English literature [11, 28]. In North America, *H. capsulatum* is endemic in the Mississippi and Ohio River valleys and also in localized foci throughout the region [11, 30, 31]. In Central and South America and the Caribbean area, it has been described in almost all countries except Chile [4, 28, 32–34]. Other endemic areas include parts of Africa, Asia (India, China, Philippines, Thailand) and Australia [7, 35–38].

In endemic areas, histoplasmosis represents the first manifestation of AIDS in up to 50–75 % of patients and occurs in about 2–25 % in HIV-infected patients living in these areas [39]. Few incidence data using cohort studies are available. In one endemic area, before the HAART era, subclinical or symptomatic histoplasmosis occurred in 12/100 person-years at risk in a cohort of HIV-infected patients [40]. Nowadays, with the advent of effective antiretroviral therapy, North American authors state that the disease is less frequently seen in HIV-infected patients in USA [11]. Still, during the HAART era, in one South American endemic area, histoplasmosis is the first AIDS-defining event with an incidence of 15,4/1000 HIV-infected person-years [41]. In disease-endemic areas where research centers and/or reference centers for the diagnosis led studies and published papers, physicians are used to seeking and treating for histoplasmosis. Thus, at the AIDS stage, histoplasmosis-related mortality is mainly around 10 % during the HAART-era [24]. But, even in high-income countries, in settings far from the reference centers’ influences and means, mortality increased up to 40 %. Apart from the host’s medical history and socio-economic factors, this observation is probably due to the lack of disease recognition and knowledge by physicians who are unable to diagnose earlier and treat a treatable disease [42].

Histoplasmosis occurs infrequently in persons living outside of, and not travelling to, endemic areas. While precise incidence figures are unavailable, the low incidence can be inferred from case reports reviews on this subject [27]. In Europe, most of these cases are considered as “imported” with exposure decades before, when staying in or travelling to a known endemic region [8–10]. Also, as described in the soil of the Po River valley in Italy, microfoci contaminated with *H. capsulatum* can be found in regions considered as non-endemic. These may be the source of exposure causing autochtonous cases described in several European countries [10]. Historically, mortality in non-endemic areas was observed at around 50 to 60 % [8, 9]; but since the availability of HAART, a marked decrease to almost 20 % was observed in some places [8].

## Pathogenesis and Risk Factors in HIV-Infected Patients

*H. capsulatum* is a thermally dimorphic ascomycete, displaying a filamentous mold form (saprophytic form) in the environment and in culture at temperatures below 35 °C, and a yeast phase (parasitic form) in tissue and body fluids at temperatures above 35 °C. The mold phase may contain macroconidia and microconidia. The latter, smooth walled with a diameter of 2 to 4 μm, are the infectious particles. The yeast phase develops as small oval budding cells with a diameter of 2 to 4 μm, mainly observed within the macrophages. In Africa, the yeast phase described is mostly thick walled and larger, 8 to 15 μm in diameter [18].

*H. capsulatum* is found in soils throughout the world. It grows best in soils with high nitrogen content, particularly those enriched with bird or bat guano. Birds do not become colonized or infected with *H. capsulatum* (since their body temperature is too high), and their droppings are primarily a nutrient source for the development of fungal pathogen. Soil samples from sites where birds have roosted have remained contaminated for at least 10 years after the roost has been cleared, even in urban areas [18, 43].

Upon disruption of soil containing the organism, infection with *H. capsulatum* mainly develops when microconidia or hyphal elements are inhaled and convert into yeasts in the lungs, or when organisms in old foci of infection reactivate during immunosuppression [44]. Once deposited in the lungs, *H. capsulatum* is internalized by resident and recruited phagocytes, including macrophages, dendritic cells, and neutrophils. The intracellular fate of the organism is divergent in these cellular populations. While neutrophils and dendritic cells have fungistatic and fungicidal activity, respectively, *H. capsulatum* survives in macrophages prior to cellular activation regulating the pH of the intraphagosomal environment. Phagocytes facilitate dissemination of *H. capsulatum* to several organs, throughout the mononuclear phagocyte system, including the spleen, liver, bone marrow, and lymph nodes. Thus, to control intracellular growth of the organism, the host must mount a robust proinflammatory response, and cellular immunity plays an essential role in defense against *H. capsulatum* [45]. Therefore, with the development of HIV-infection and the progressive impairment of cellular immunity, patients are at a higher risk of disseminated and deadly infections with *H. capsulatum*.

In endemic areas, whether symptomatic diseases are caused by reactivation of old foci or by a recent exogenous exposure is unknown. Published data support reinfection in the context of outbreaks or reactivation when calcified lymph nodes are visualized on chest radiograms [39]. Recently, a time series attempted to estimate if new infection is prevailing over reactivation in endemic areas. Using climatic data, in one endemic area, 70 % of incident HIV-associated histoplasmosis cases could be predicted compared to observed cases [44]. Also, a clear seasonality pattern in incident cases of histoplasmosis was described [46]. These studies are in favor of new infection or reinfection rather than reactivation as the main pathogenesis mechanism to explain the occurrence of symptomatic histoplasmosis in endemic areas [44, 46].

Among Risk Factors Previously Studied in HIV-Infected Patients, Occupational or Environmental Factors and Host Factors Have Been Described

Environmental exposures to sources containing bat or bird guano are known to increase the risk of histoplasmosis in the general population, including histories of cave exploration, wood cutting, or exposure to bird roosts, excavation sites, farms or chicken coops [47]. In HIV-infected individuals experiencing histoplasmosis, prospectively studied and compared to control without histoplasmosis, a history of exposure to chicken coops was found to be significantly associated with histoplasmosis [47].

Current or prior occupations or activities with soil contaminated with bird or bat droppings have been described to be associated with an increased risk of histoplasmosis [40, 48].

Host factors previously found to be independently associated with histoplasmosis are: low CD4 count (<200/mm^3^), low CD4 count at the NADIR (<50/mm^3^), low CD8 count (<650/mm^3^), absence of antiretroviral treatment or the first 6 months under antiretroviral therapy, a history of herpes simplex infection, absence of systemic antifungal therapy (fluconazole) and male gender [40, 49].

Out of *H. capsulatum* transmission to recipient by donor's solid organ transplant, no direct interhuman contamination has been previously described [50].

## Clinical Findings

Although it is mostly asymptomatic and self-limiting in immunocompetent persons, histoplasmosis in immunocompromised HIV-infected patients presents as a symptomatic and disseminated infection in 95 % of the cases [11]. With the aggravation of CD4 Lymphocyte decline, the evolution is rapid and always fatal in the absence of treatment [2]. Thus, according to published series, there is up to 39 % death following diagnosis in endemic areas and 58 % death in non-endemic areas [9, 24].

During the HIV infection, the evolutive mode is very variable, from extreme latency to a fulminant form. For 10–20 % of patients, severe rapidly fatal forms are described. These forms present as a septic shock with intravascular disseminated coagulation, multiorgan failure (kidneys, liver, lungs), and rhabdomyolysis, all of which may be associated with a hemophagocytosis syndrome. These presentations of unclear pathogenesis seem to be a late manifestation of histoplasmosis [39]. Mortality of these severe cases is very high, with 50–70 % death [51].

Inflammatory reconstitution disease following antiretroviral treatment initiation has also been described [8, 52].

All organs and tissues may be clinically involved [11]. In HIV-infected patients histoplasmosis is disseminated in 95 % of the cases, and in 90 % of the cases, it concerns patients with CD4 counts below 200/mm3. A subacute presentation is the most frequent, with symptoms evolving for 1 or 2 months, during which the patient is seen by physicians. The clinical picture is misleading, the symptoms being mostly nonspecific. The general symptoms, particularly fever, fatigue and weight loss, are almost always found. Respiratory symptoms such as cough or dyspnea are observed in 50 % of the cases, and may be associated with hepatosplenomegaly (25 % of cases) and/or superficial lymph node enlargement (25 % of cases). Digestive, neurological or muco-cutaneous manifestations are less frequent (10–20 % of cases) and polymorphous. Isolated pulmonary manifestations have also been described in less immunocompromised patients (CD4 > 200). Pulmonary intersticial syndromes are often observed on chest radiograms or computed tomography (CT) scans. This implies that bronchoscopy must be performed to rule out the differential diagnosis of pneumocystosis [39].

Similarly to clinical symptoms, standard biology tests are nonspecific. However, they may give elements to suspect the diagnosis and lead to further investigations to confirm the diagnosis. The elevation of lactate dehydrogenase (LDH), liver enzymes (TGO, alkaline phosphatase), ferritin alone or isolated with pancytopenia and/or hemophagocytosis syndrome, are classically described [39].

Thus, a high suspicion index is required from clinicians because of the nonspecific nature of the clinical, biologic and radiologic spectrum of histoplasmosis. In disease-endemic areas, this nonspecific clinical picture makes tuberculosis the main differential diagnosis of histoplasmosis. Numerous publications report cases of histoplasmosis mimicking tuberculosis and state that making this differential diagnosis at the bed patient is difficult; mainly because of the absence of diagnostic facilities or because a diagnosis of histoplasmosis was not considered. Nevertheless, tuberculosis and histoplasmosis could be easily distinguished, as reported recently. Numerous AIDS-related death caused by histoplasmosis and mistaken for multi-drug-resistant tuberculosis could be avoided [53].

## Diagnosis

Direct examination of MGG-stained slides of all tissue or body fluid is a simple and rapid diagnostic method. In disseminated forms, smears obtained from tissue biopsies, bone marrow aspiration, or peripheral blood are fairly contributive. However, sensitivity varies according to the type of sample, the severity of the disease and the experience of the operator. With positive results for 50 to 75 % of patients, bone marrow aspiration is the most contributive [24, 39]. Special staining (PAS and Gomori Grocott) may be performed in pathology. These stainings allow one to rule out the main differential diagnoses, such as *C. glabrata*, *P. marneffei*, *Leishmania*, *Trypanosoma* and other staining artifacts [24].

Certain diagnosis rests on the culture and identification of *Histoplasma capsulatum* from any tissue or body fluid, obtained mainly by using invasive procedures. The isolation in culture is slow and may take several weeks (1–6 weeks). Sabouraud’s dextrose agar media are thus incubated for several weeks between 25 °C and 30 °C. The observation of macronidia and micronidia are characteristic, but since they are highly infectious, this must be performed in a Biosafety Laboratory (BSL) level 2 or a BSL 3, according to the country’s regulations. A definitive, specific and commercially available DNA probe assay should always be performed to confirm the diagnosis. The laborious task of converting the mold phase to the yeast phase is no longer required for the definitive identification of *H. capsulatum* [11].

Specificity is 100 %, but sensitivity varies between 85 and 90 % according to the fungal load and the laboratory experience [24, 54, 55]. Bone marrow aspirates yield the highest proportion of positive cultures (70–90 %). For peripheral blood culture, authors notably recommend the lysis-centrifugation technique, in order to increase sensitivity and to reduce the identification delay relative to other techniques [39]. For the diagnosis of isolated neuro-meningeal forms, the diagnosis must rely on other techniques, given the very low sensitivity of cerebrospinal fluid (CSF) culture [54].

Antibody detection by immunodiffusion or complement fixation is less sensitive in immunocompromised HIV-infected patients than in immunocompetent patients (90 %) [39, 54]. Serology is only positive in 50–70 % of immunocompromised HIV patients [39, 54]. The rise of antibody titers is observed 2–6 weeks after exposure, and the lack of discrimination between active and passive infection are not compatible with the management of acute cases. However, serology on CSF may be of interest for the diagnosis of neuro-meningeal forms [56].

Immunodiffusion is more specific but less sensitive than complement fixation. Cross-reactions with other fungal pathogens, lymphoma, sarcoidosis and tuberculosis have been reported [57].

The detection of *Histoplasma capsulatum* var. *capsulatum* circulating antigen may be performed with several EIA methods.

The third generation polyclonal MVista *Histoplasma* antigen EIA allows the quantitative detection of *Histoplasma* polysaccharide circulating antigen with a sensitivity of 95–100 % in urine and 92–100 % in serum [55, 58]. The antigen levels detected are higher in the immunocompromised patients with a disseminated form. Moreover, the antigen level seems correlated with the severity of the clinical presentation (admission or not in ICU) [55]. Specificity is 99 %, and positive and negative predictive values are 91 % and 99.5 %, respectively (for a 10 % prevalence) [58]. In pulmonary forms of histoplasmosis, this test may be performed on bronchioloalveolar lavage fluid with a 93 % sensitivity [59]. Despite the evolution of the test, there are still cross–reactions with blastomycosis (90 %), paracoccidioidiomycosis or penicilliosis (80 %), coccidioidiomycosis (60 %), aspergillosis (10 %) and sporotrichosis [55, 60]. However, this method is currently the best validated method for the diagnosis of histoplasmosis in HIV-infected patients. Not commercialized, it is hardly used out of the USA [24, 61].

The IMMY ALPHA *Histoplasma* antigen EIA has been marketed since 2006. Sensitivity is 71 % and specificity is 98 % in urine. Using the same detection and antigen capture technique with polyclonal antigens, similar cross-reactions are observed with fungal pathogens (Paracoccidioidiomycosis, coccidioidiomycosis and blastomycosis) [62]. This first test seems less utilized, having generated debates in the literature [62–64]. However, recent modifications and the development of monoclonal antibodies seem very promising [65, 66].

The Mycotic Disease Branch of the Centers for Disease Control and Prevention (CDC) also developed an EIA *Histoplasma* antigen detection method with a sensitivity of 81 % and a specificity of 95 % in the urine of HIV patients in Guatemala. Cross-reactions were only observed for paracoccidioidiomycosis [67]. This test is routinely used in Colombia and is currently being evaluated on the Guiana Shield (ClinicalTrials.gov Registration number: NCT01884779).

Since MVista test can’t be performed out of the MVista laboratory in Indianapolis, the IMMY and CDC EIA tests developments are of importance, because they are designed to improve methods for the detection of *Histoplasma capsulatum* in regions with limited resources where the organism is endemic and where delayed diagnosis of histoplasmosis results in high mortality rates.

False positives have been observed with the Platelia *Aspergillus* EIA (BioRad) in the serum of 50–70 % of patients with confirmed histoplamosis [68–71]. This cross-reaction may be of interest in hospitals that do not have access to *Histoplasma* EIAs, by taking into consideration the epidemiologic context and other potential causes of false positives in the Platelia *Aspergillus* test [71].

The molecular diagnosis by Polymerase Chain Reaction (PCR) gives rapid results with good sensitivity and specificity in tissues and body fluids of HIV-infected patients [72, 73]. However, its place in patient care is not clear, since it has not been externally validated and none of the methods have been commercialized [57, 74]. Recently, a loop-mediated isothermal amplification (LAMP) assay, a potential inexpensive point of care diagnostic tool, proved the concept that the assay can be used to detect *Histoplasma* DNA in urine. Still, further evaluation of this assay using body fluid samples from a larger patient population is warranted [75].

## Treatment

The treatment of *Histoplasma capsulatum var. capsulatum* infection in HIV-infected patients has led to recommendations in the USA and in France [76, 77]. However, there are few recent data based on randomized controlled trials: the 2007 North American recommendations are still valid [78, 79].

While waiting for mycological confirmation of the diagnosis of a patient with a strong suspicion of histoplasmosis with or without severity symptoms, clinicians have two options for the treatment induction: intravenous (IV) amphotericin B or oral itraconazole. Although amphotericin B is fungicidal and has shown its efficacy in terms of survival, it is also nephrotoxic [80]. Itraconazole is fungistatic and is associated with drug interactions that complicate patient care in the context of profound immunosuppression. This, added to the diminished bioavailability of itraconazole in HIV patients, makes the recommended serum concentration level long and difficult to reach.

In a randomized clinical trial, IV liposomal amphotericin B was more effective than deoxycholate amphotericin B, with a quicker clinical response, a decreased toxicity, and a decreased mortality [80]. Thus, for the induction treatment of moderately severe and severe presentations of disseminated histoplasmosis, liposomal amphotericin B (3 mg/kg/day) is the recommended strategy for 2 weeks or until clinical improvement. A relay with oral itraconazole (200 mg three times a day for 3 days, then twice a day) must then be initiated for at least one year [78].

In order to avoid overlooking presentations with high short-term lethality, and to better guide treatment choice, studies were led to identify risk factors associated with severe histoplasmosis [40, 51]; these serve as the basis for the 2004 definition of histoplasmosis severity by the CDC [81]. Severe cases of histoplasmosis were defined as patients presenting one or more of the following criteria: temperature >39 °C, systolic blood pressure <90 mmHg, arterial oxygen pressure <70 mmHg, weight loss greater than 5 %, a Karnofsky score <70, haemoglobin concentration <10 g/dL, neutrophil counts <1000/mm^3^, platelet count <100 000/mm^3^, aspartate aminotransférase >2.5 times the normal threshold, serum bilirubine or creatinine exceeding twice the normal threshold, albumine concentration < 3.5 g/dL, the presence of a coagulopathy, the presence of another organ dysfunction or confirmed meningitis [81]. This definition seems scarcely used in routine, and no severity score is available to guide clinicians in their therapeutic choices.

In severe forms of histoplasmosis, renal failure is often described: either associated with multiorgan failure, or secondary to amphotericin B nephrotoxicity. Thus, clinicians tend to switch to itraconazole in order to not aggravate renal failure. However, the poor prognosis of these clinical presentations justifies the continuation of the most effective drug, amphotericin B, despite its nephrotoxicity. In most cases, renal function is restored *ad integrum,* despite the use of this nephrotoxic therapy [78].

For non-severe cases, itraconazole is the first-line treatment with the same protocol as above. The response is positive in 85 % of the cases [82].

The surveillance of itraconazole concentrations is recommended, notably when drug interactions are suspected (particularly with protease inhibitors, efavirenz or rifampicine). The surveillance of serum concentrations must take place 7–15 days following initiation. On a random blood sample, the serum concentration of itraconazole must be >1 μg/mL [78]. In terms of intestinal absorption, the itraconazole syrup formulation, prescribed in a fasting patient, seems preferable and better tolerated than capsules prescribed during a meal [76].

The evaluation of treatment efficacy with the regular monitoring of serum and urine *Histoplama* antigen concentrations using MVista *Histoplasma* antigen EIA is only available and recommended in the USA [76, 83]. For countries where this test is not available, under some conditions, notably the knowledge of the local epidemiology of cross-reacting fungal infections, certain authors consider the use of the Platelia *Aspergillus* EIA (BioRad) to monitor the therapeutic efficacy of antifungals [68, 71].

Acute non-disseminated pulmonary presentations in HIV-infected patients with CD4 counts >300/mm^3^ are treated like immunocompetent individuals [78].

Confirmed neuro-meningeal HIV-associated histoplasmosis is initially treated with IV liposomal amphotericin B (5 mg/kg/day) for 4–6 weeks. A relay with oral itraconazole 200 mg two to three times per day is instated for at least one year, and until the normalization of the CSF abnormalities [78].

Oral posaconazole and voriconazole are considered second-line treatments for non-severe disseminated histoplasmosis.[76]. Fluconazole is less effective than itraconazole, but may be used as a second-line treatment in itraconazole-intolerant patients at a dose of 800 mg/day [84].

Antiretroviral treatment must be introduced rapidly following the initiation of the antifungal treatment. Immune reconstitution inflammatory syndromes have been described, but are mostly non-severe and are not considered sufficient to warrant delaying antiretrovirals to restore cellular immunity, the key defense against *Histoplasma capsulatum* infection [76, 78].

## Prevention

Maintenance therapy (secondary prophylaxis) uses oral itraconazole 200 mg/day. It must be continued for life if the immunosuppression persists or if histoplasmosis relapses despite an appropriate treatment. Primary prophylaxis with itraconazole (200 mg daily) is recommended in HIV-infected patients with CD4 cell counts <150 cells/mm3 in specific areas of endemicity where the incidence of histoplasmosis is >10 cases per 100 patient-years [78]. The USA is the only endemic area recommending primary prophylaxis against histoplasmosis in immunosuppressed individuals, a strategy that has recently been shown to be cost-effective, particularly in low and middle income settings [85].

To our knowledge, the USA is also the only one to edit detailed recommendations regarding occupational or environmental exposure to protect workers at risk of histoplasmosis [86].

Recently, concerns about immunosuppressed travelers have been published. These patients represent an increasing group of travelers, for business or tourism. Those with severe cellular immunodeficiency, like advanced HIV infection, display the highest risk of fungal infections. Thus, a systematic visit in a travel clinic for immunocompromised patients traveling to the tropics ensures that the specific risks of acquiring fungal infections (and others) are understood. When immunocompromised hosts return to their area of residence, a nonbacteriologically documented, potentially severe, febrile pneumonia, with or without dissemination signs (skin lesions, cytopenia) should alert for travel-acquired fungal infection, even years after return. Localized subcutaneous nodule may be also ascribed to fungal infection. Finally, infectious disease physicians should be aware of major clinical patterns of travel-acquired fungal infection, as well as the fungi involved, and risk factors according to the geographical area visited [87].

## Conclusion

Nowadays, the real burden of histoplasmosis is not known, it is only based on estimates and not on hard data. Histoplasmosis is still a public health problem during the HAART era, notably in HIV-infected individuals. The medical mycological iceberg statement, written 40 years ago, is still ongoing throughout the world [29]. Outside of research centers or reference centers for the diagnosis of histoplasmosis, in most of the countries where it is endemic, the disease remains either mistaken for tuberculosis or neglected. There are still research needs and accomplishments to target in order to better understand, diagnose and treat individuals infected with *H. capsulatum*. “*Know your epidemic, know your response*”; it is time to act against histoplasmosis [15, 17].
